# An innovative destressing technology and key parameters determination in both sides of a deep roadway

**DOI:** 10.1038/s41598-023-32015-7

**Published:** 2023-03-23

**Authors:** Zaisheng Jiang, Dongdong Chen, Shengrong Xie

**Affiliations:** 1 School of Energy and Mining Engineering, China University of Mining and Technology-Beijing, Beijing, 100083 China; 2 Beijing Key Laboratory for Precise Mining of Intergrown Energy and Resources, China University of Mining and Technology-Beijing, Beijing, 100083 China

**Keywords:** Energy science and technology, Engineering

## Abstract

The general or single supporting theory and technology of the shallow surrounding rock of the roadway are not suitable for solving the problem of continuous large deformation of the both sides under the continuous migration of coal mass in the deep domain of the roadway side. Furthermore, the general destressing technology of dense drilling in the roadway destroys the shallow anchorage domain while releasing the stress. Therefore, this study proposes the “shallow supporting and deep destressing” synergism technology. This technology provides puissant supporting in the shallow domain of roadway sides, and at the same time, large destressing holes are excavated at the coal mass migration channel in deep stress peak domain far from the anchorage domain, conducting destressing regulation of roadway sides. This technology can shift stress peak domain of the roadway side to solid coal side of destressing hole without destroying the shallow anchorage domain, and at the same time, provide a buffer space for that coal mass in the deep domain of the roadway side continuously migrates to the anchorage surrounding rock, creating a beneficial stress circumstances for the roadway stability. The “shallow supporting and deep destressing” synergism technology can solve the contradiction between the shallow surrounding rock supporting and the continuous migration of coal mass in deep domain. The field application results show that the innovative destressing technology can effectively solve the problem of surrounding rock control in deep roadway.

## Introduction

As a necessary channel for the exploitation of coal resources, the stability and safety of coal mine roadways is fundamental to realizing the safe and efficient production of deep mine^1–4^. With the depletion of shallow coal resources, deep mining has gradually become the norm of coal resources development^5,6^. The mechanical characteristics of the deep coal and rock mass cause the surrounding rock deformation of deep roadways exhibit strong ground pressure characteristics such as large deformation, fast displacement convergence rate, and damage to the support system^7–10^. This is because after the coal mine enters the deep mining, the coal and rock mass are under the action of high stress, high temperature, high permeability and strong time effect for a long time, which leads organizational structure, foundation mechanical behavior characteristics and engineering response of coal mass have undergone fundamental changes^11–14^. The deep roadway is often repaired many times and still cannot meet the section requirements required for safe production of the mine, which seriously restricts the safe and efficient mining of deep coal resources^15–18^. In order to solve the problem of maintaining the stability of deep roadway, scholars have conducted a lot of studies and pointed out that the improvement of surrounding rock stress, the grouting modification of surrounding rock and the strong support of surrounding rock are the main factors affecting the stability of deep roadway^19–22^. It is difficult to establish a strong support system or to study the modification mechanism of the surrounding rock and the properties of grouting materials to control the large deformation of deep roadways, but improving the stress state of the surrounding rock is fundamental to maintaining the stability of a deep roadway^23,24^. Therefore, research on destressing technology for roadways such as drilling, opening slots, and loose blasting has been carried out, and significant progress has been achieved.

Wang et al. systematically studied the effect drilling, to relieve pressure, on the stability of the surrounding rock of a deep roadway and proposed creep control countermeasures for them^25,26^. Zuo et al. founded an equivalent elliptical mechanics model for opening slot destressing, revealed the mechanism of opening slot destressing, and obtained the optimal scheme for the same^27^. Jia et al. studied the effects of drilling diameter, spacing, and drilling depth parameters on the strength of the sample through laboratory tests and analyzed the failure modes of samples under the influence of different parameters^28^. Zhang et al. concluded that the larger the drilling density, greater was the rock fracture development around the boreholes, and more effective the relief effect^29^. Zhang et al. studied the angles of elastic energy dissipation, stress transfer, the distribution law of the destressing area, and the areas of stress concentration, energy, stress, displacement, and plastic behavior of large diameter destressing boreholes in coal seams under high-stress conditions^30^. Li et al. analysed the destressing magnitudes in coal mass around the boreholes under different diameters, spacing, and drilling time and monitored the borehole destressing in a coal mine on-site^31^.

**Figure 1:**
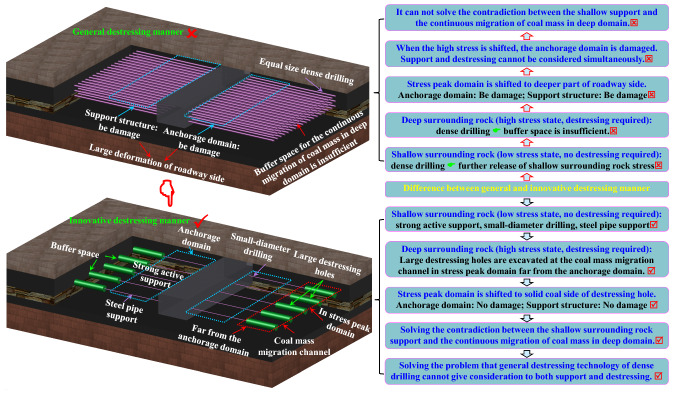
Difference between general and innovative destressing manner.

Although the above work made some useful conclusions relating to the application of destressing technology and parameter determination, their researches had the following limitations. (1) Their studies treat the surrounding rock deep part and shallow part equally, simply drilling dense, equal size, large holes on roadway both sides. (2) Owing to the crush of shallow surrounding rock, the shallow surrounding rock has been in a low-stress state. So, the shallow part surrounding rock does not require destressing. (3) Owing to drilling dense, equal size, large holes on roadway both sides simply, the coal mass and support architecture of shallow surrounding rock will be more damaged when migrating stress by dense drilling destressing. In view of this, this study proposes the “shallow supporting and deep destressing” synergism technology to overcome the above three defects. This technology has the following characteristics different from traditional destressing methods: (1) Distinguish the shallow part of the roadway sides from the deep part. (2) Non-intensive small holes are arranged in the shallow surrounding rock of the roadway side; large destressing holes are excavated at the coal mass migration channel in deep stress peak domain far from the anchorage domain. (3) Stress peak domain of the roadway side is shifted to solid coal side of destressing hole without destroying the shallow anchorage domain. Difference between general and innovative destressing manner as is shown in Fig. 1.

## Project overview

### Engineering geology circumstances

The panel corresponding to the test roadway is 2-2-1 panel. The roadway is mainly affected by the dynamic pressure of 2-2-1 panel. The design average strike length of the 2-2-1 panel is 1127 m, and the average slope length is 226 m. The average thickness of coal seam in the 2-2-1 panel is 5.4 m. The recoverable reserves in the 2-2-1 panel are 1.793 million tons. The immediate roof is 2.32-m-thick siltstone with dense and uniform lithology. The main roof is 8.9-m-thick fine sandstone with dense and hard lithology. The floor is 1.06-m-thick fine sandstone with argillaceous cementation and horizontal bedding.

**Figure 2:**
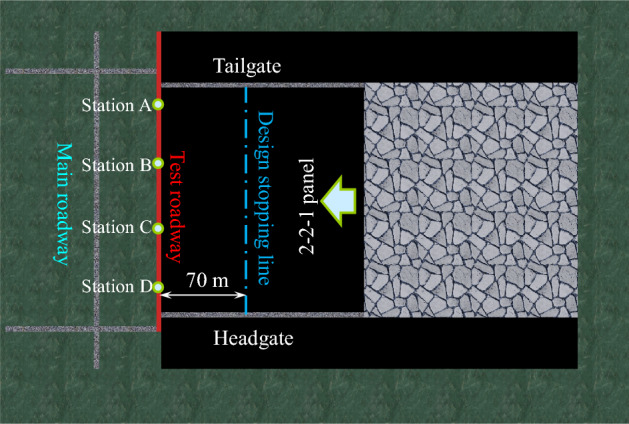
Roadway layout.

The test chamber, a rectangular large-section coal roadway (width × height = 5.0 × 3.0 m), was arranged between the 2-2-1 panel and the main roadway. Joints and fissures in the coal body near the test coal roadway are clearly developed. The test roadway was approximately 70 m from the stopping line of the 2-2-1 panel. The roadway was excavated along the roof of the 2# coal seam, and the buried at a depth of approximately 660 m. The layout of the roadway is shown in Fig. 2.

**Figure 3:**
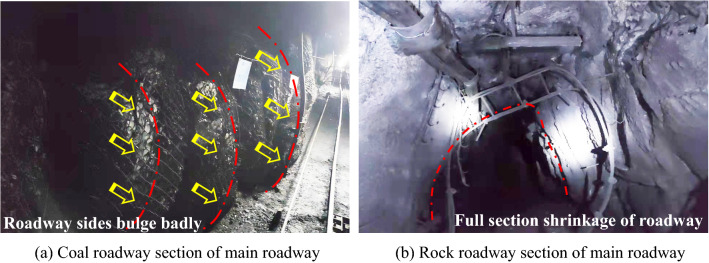
Large deformations and failures for main roadway.

The distance between the main roadway and the stopping line of the 2-2-1 panel exceeded 140 m. Before the mining of the 2-2-1 panel, the main roadway suffered from large deformations and failures many times after being impacted by the mining turbulence of the other adjacent panel (see Fig. 3).

### Turbulence influence degree of 2-2-1 panel

**Figure 4:**
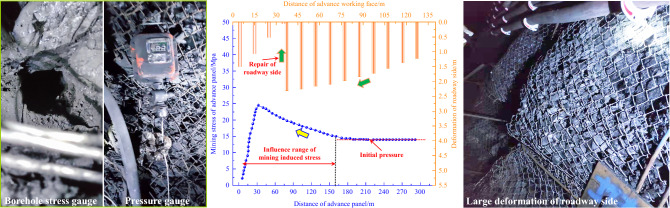
The influence range of mining induced stress in the panel and the deformation of both sides of the mining roadway.

Three measuring stations (1 #, 2 # and 3 #) are set at the solid coal side of the mining roadway in front of the advanced panel. Each measuring station is equipped with two drilling stress meters. A total of six drilling stress meters are arranged. The 1 # measuring station is located at 200 m of the advanced panel, the 2 # measuring station is located at 400 m of the advanced panel, and the 3 # measuring station is located at 600 m of the advanced panel. The spacing between two adjacent drilling stress meters in the same station is 2 m. The diameter of each drilling is 45 mm, the depth of the drilling is 13 m, the drilling stress meters is about 1.2 m from the roadway floor, and the drilling is arranged perpendicular to the coal wall. Monitor the change laws of stress monitoring data of drilling stress meter as the mining face approaches the above stations. The drilling stress meter arranged at 600 m in the roadway of the advanced panel can not only monitor the influence range of the mining stress of the panel, but also verify the rationality of monitoring data of the drilling stress meter at the 1 # and 2 # measuring stations. Then draw the corresponding law between the stress and the leading distance of the panel, and get the influence range of the mining stress of the panel. Also, the displacement monitoring points are arranged to monitor the deformation of both sides of the mining roadway of advanced 2-2-1 panel. The results are shown in Fig. 4 below:

In the panel mining process, when the measuring point is 160 m away from the panel, the stress value almost remains unchanged; when the measuring point is within 160 m of the panel, the stress begins to increase gradually, entering the influence domain of mining induced stress of the panel. It can be seen that the influence range of mining induced stress in the panel can reach about 160 m in front of the panel.

It can be seen from the both sides deformation of the mining roadway that the deformation of both sides of the mining roadway within 125 m in front of the panel is more than 1 m, and the both sides of the roadway about 37 m in front of the panel have been repaired, but the deformation of the surrounding rock of both sides after expanding is still increasing. It can be seen that when the 2-2-1 panel is approaching the stoping line, the test roadway 70 m away from the stoping line is within the puissant mining range of the panel, which will seriously threaten the safety and stability of the test roadway.

### Deformation characteristics of test roadway

**Figure 5:**
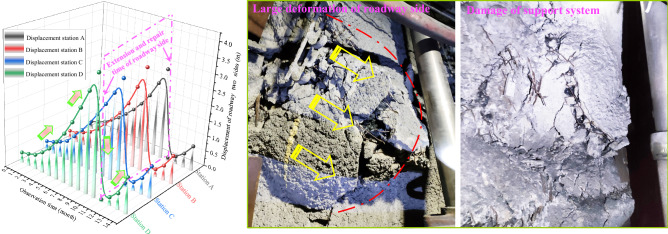
Deformation curve of each measuring station without panel advance turbulence and deformation on site.

When the test roadway was not impacted by the turbulence of the 2-2-1 panel, the both sides underwent a continuous large deformation. Figure 5 shows the curve indicating change in displacement for the both sides of the test roadway. An analysis of Fig. 5, revealed that when the test roadway was not impacted by the mining turbulence of the adjacent 2-2-1 panel, the deformation of the roadway surrounding rock increased continuously. Roadway need to be expanded and repaired every year.

The field measurements show that the displacement of the both sides of the expanded and repaired roadway continued to increase, and the deformation rate gradually increased. Therefore, advancing the 2-2-1 panel near the stopping line would cause severe turbulence to the test roadway. Under the influence of a severe turbulence, large scale deformation and damage, and catastrophic accidents could inevitably occur in the test roadway.

### Previous supporting mode and roadway failure

As the surrounding rock of the test roadway has to be expanded and renovated regularly, the combined control technologies of active puissant supporting and grouting modification were adopted in the roadway. Previous supporting parameters are shown in Table 1.

**Table 1:** Previous supporting parameters.

	Roof grouting cables	Roof bolt	Sides grouting cables	Sides bolt	Single hydraulic prop
Dimension	φ21.8 × 10,500 mm	φ22 × 2200 mm	φ21.8 × 6500 mm	φ22 × 2200 mm	–
Spacing	2.4 × 3.2 m	0.8 × 0.8 m	1.2 × 1.6 m	0.8 × 0.8 m	–
Specifications	–	–	–	–	Each row two props
Supporting facilities	–	–	–	–	Supporting with a π steel beam
Row spacing	–	–	–	–	1.0 m

**Figure 6:**
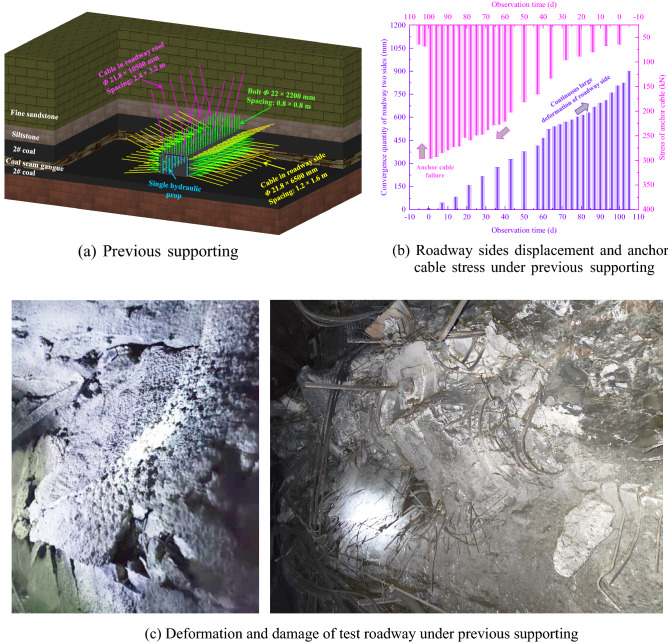
Previous supporting mode and roadway failure.

After the above puissant supporting technologies (see Fig. 6a), were adopted in the roadway, the displacement and anchor cable stress observed in the both sides of the chamber are shown in Fig. 6b; the deformation and damage of test roadway under previous supporting is shown in Fig. 6c. Therefore, it is clear that continuous deformation of the surrounding rock continues to occur even after adopting general active puissant supporting and grouting technology.

It can be concluded that when the 2-2-1 panel is advanced to the stopping line, the chamber would be suffered from a severe turbulence of the 2-2-1 panel, resulting in a wider extent of damage. Therefore, effective control measures must be taken to prevent the large-scale deformation of the surrounding rock the both sides of the chamber before the 2-2-1 panel is advanced to the stopping line.

### Main control difficulties of surrounding rock of test coal roadway

1. Deep complex geological conditions.

The deep rock mass, with strong time effect, is located in the complex environment of high stress, high temperature and high karst water pressure. Mechanical properties of deep rock mass is manifested by obvious rheology or creep, and is prone to large range of non-steady and nonlinear rheological phenomena, resulting in the failure of roadway support structure and continuous large deformation. That makes the difficulty of surrounding rock control of coal roadway be increased.

(2) Strong mining influence of the panel.

The mining influence of panel is wide and strong. In addition, the deep coal and rock mass are in the environment of high stress, high temperature and high karst water pressure. The superposition of high in-situ stress and mining stress leads to the prominent dilatability and rheology of surrounding rock deformation. The surrounding rock of the roadway is prone to unstable failure characteristics such as continuous large deformation, and may cause disasters in serious cases.

(3) Large cross section increases the difficulty of surrounding rock support in coal roadway

Due to the large section of the roadway and the soft coal mass, the crack development and extrusion deformation of coal roadway side are serious after the roadway excavation and support. Especially for the ordinary bolt (cable) support, when the strength of the anchorage bearing structure is low or the support structure is damaged, the surrounding rock deformation will rapidly increase, affecting the safety of the roadway surrounding rock support.

## Numerical analysis

### Numerical calculation model

**Figure 7:**
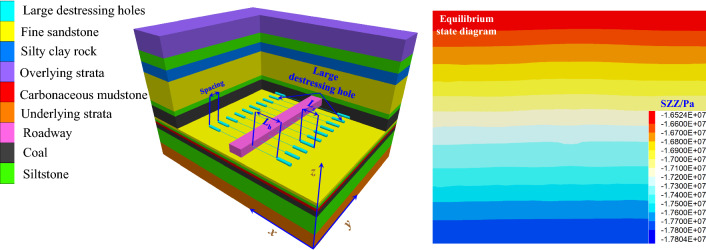
Numerical calculation model.

1. Establishment of numerical model:

According to the geological conditions, the direction of extension of the large destressing hole was the x-axis (80 m), the axial direction of the chamber was the y-axis (100 m), and the vertical direction of the coal and rock mass was the z-axis (80 m), forming a numerical calculation model as shown in Fig. 7. The velocity of the front, rear, left, right and bottom boundaries of the model is 0. The vertical stress of 16.5 MPa is applied to the top boundary of the model to simulate the weight of the overlying strata. The lateral pressure coefficient of the model is taken as 1.2. The numerical calculation model systematically studied the laws governing the different large destressing hole parameters (large destressing hole place, large destressing hole length, and large destressing hole spacing) on the destressing effect of the surrounding rock of a deep roadway. It provides guidance for determining the parameters of a large destressing hole on-site. The deformation and failure of the surrounding rock mass of the coal roadway obey the Mohr–Coulomb calculation criteria.

The mechanical parameters of the rock stratum are listed in Table 2.

**Table 2:** Actual physical and mechanical properties of each stratum.

Lithology	Density/kg m^−3^	Bulk modulus/GPa	Shear modulus/GPa	Internal friction angle/°	Cohesion/MPa	Tensile strength/MPa
Silty clay rock	2150	6.55	5.3	29	2.8	2.0
Fine sandstone	2590	6.98	5.3	36	3.4	2.5
Siltstone	2602	7.11	6.3	35	3.0	2.1
2# coal	1400	2.6	1.5	18	0.8	0.4
Carbonaceous mudstone	2200	7.5	6.3	29	2.9	2.2
Coarse sandstone	2650	9.5	7.3	32	3.1	2.4

(2) Numerical simulation scheme:

To study the dynamic response characteristics of stress, in the surrounding rock of the test roadway, under different large destressing hole parameters, an orthogonal analysis of the numerical simulation of the factors affecting the destressing effect was carried out (37 numerical models in total), as shown in Table 3.

**Table 3:** Orthogonal numerical simulation scheme.

Large destressing hole place/m	Large destressing hole length/m			
0	–	–	–	–
4	2	3	4	5
6	2	3	4	5
7	2	3	4	5
8	2	3	4	5
9	2	3	4	5
10	2	3	4	5
11	2	3	4	5
12	2	3	4	5
13	2	3	4	5

In addition, based on the above simulation, a single factor analysis of the numerical simulation of the large destressing holer spacing was carried out, and the simulation schemes under different conditions were set (a total of five numerical models), as shown in Table 4.

**Table 4:** Simulation scheme under different spacing conditions.

Scheme number	Large destressing hole spacing/m	Large destressing hole place/m	Large destressing hole length/m
1	2.0	10.0	5.0
2	3.0	10.0	5.0
3	4.0	10.0	5.0
4	5.0	10.0	5.0
5	6.0	10.0	5.0

### Evaluation index of destressing effect

**Figure 8:**
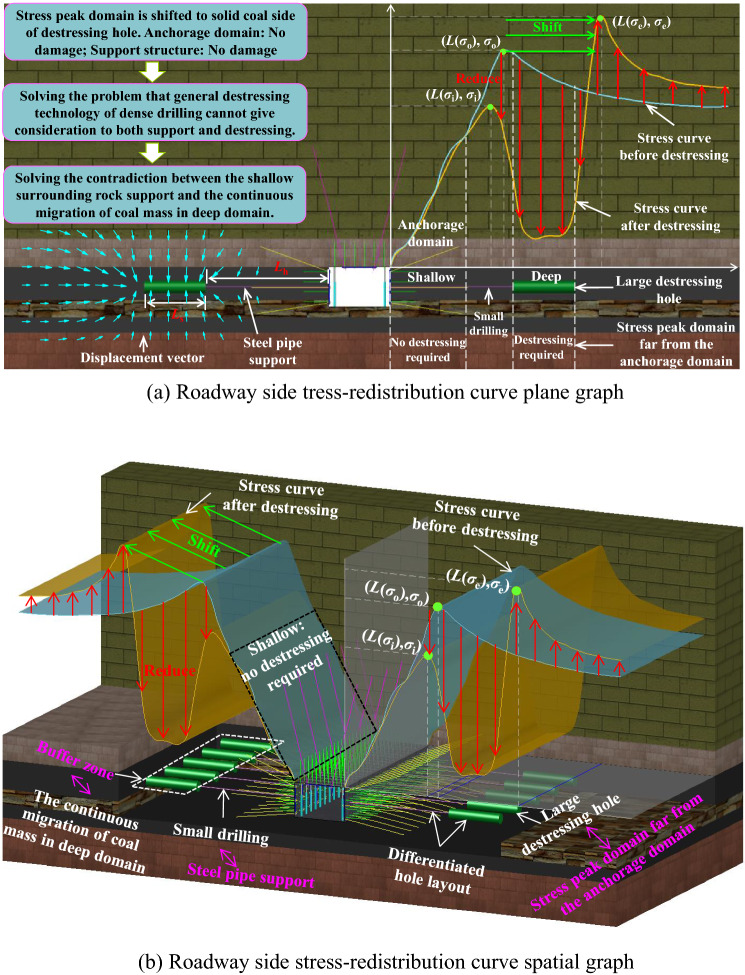
Roadway side tress redistribution curve.

The essence of innovative destressing technology is to shift stress peak domain of the roadway side to solid coal side of destressing hole without destroying the shallow anchorage domain, by excavating large destressing holes at the coal mass migration channel in deep stress peak domain far from the anchorage domain. Once the large destressing hole is completed, the stress on the roadway sides will be arranged in the manner of Fig. 8.

Eight parameter indexes were established to evaluate the destressing effect as follows:

① *L*_h_ − *L*(*σ*_o_): Distance from large destressing hole place to previous stress peak place.
② *σ*_i_/*σ*_r_: Specific value for the first stress peak to in-situ stress.
③ *σ*_i_/*σ*_o:_ Specific value for first stress peak to previous stress peak
④ *L*(*σ*_i_) − *L*(*σ*_o_): Distance from first stress peak place to previous stress peak place.
⑤ *σ*_e_/*σ*_o_: Specific value for second stress peak to previous stress peak
⑥ *L*(*σ*_e_) − *L*(*σ*_o_): Distance from second stress peak place to previous stress peak place.
⑦ ∇(*σ*_e_): Increase rate of second stress peak.
⑧ ∇[*L*(*σ*_e_) − *L*(*σ*_o_)]: Change rate of place shift of second stress peak.

This study comprehensively evaluates the shift effect of the high stress domain around the roadway by analyzing the variation law of the above evaluation indexes under different large destressing hole parameters.

### Determination of key parameters of large destressing hole

#### Determination of large destressing hole place

1. Comparative analysis of the influence of large destressing hole place on the destressing effect using stress nephogram

**Figure 9:**
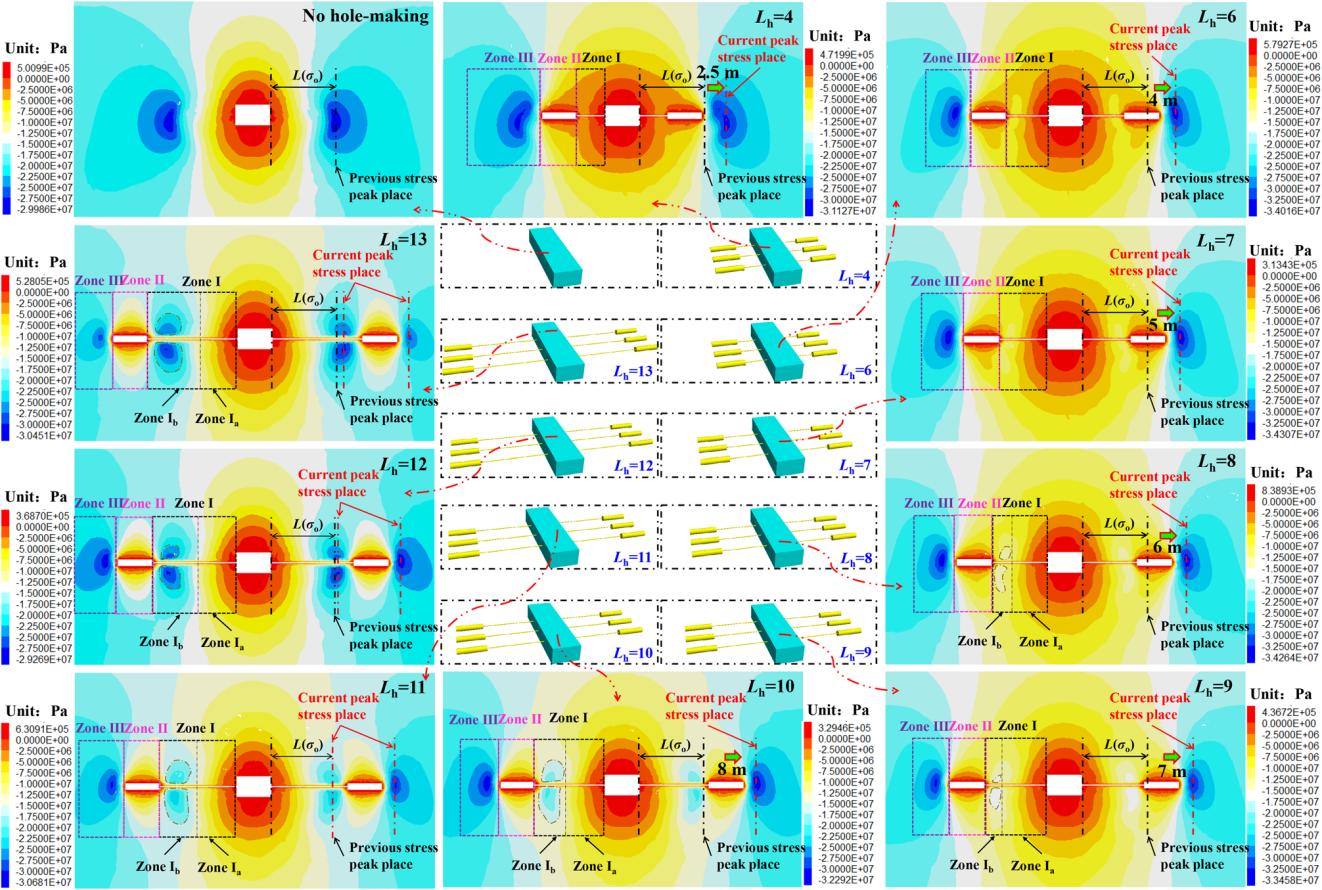
Nephogram of stress distribution of chamber surrounding rock at different *L*_h._

The stress nephogram of the surrounding rock under different large destressing hole places is shown in Fig. 9.

It can be seen from Fig. 9 that after placing the large destressing hole is completed, the stress in the surrounding rock, on the both sides of the roadway, is constantly adjusted by changing the large destressing hole place, as follows:

① After the large destressing hole is completed, the roadway sides can be divided into three zones according to the stress redistribution state of the roadway sides and the attribute characteristics of the surrounding rock: anchorage bearing zone (zone I), buffer zone (zone II), and high stress migration zone (zone III). Bolts (cables) are applied to the coal mass in zone I to form a compression arch bearing structure on the both sides of the roadway. The bolts (cables) and surrounding rock in the bearing structure bear together to resist the joint action of the dynamic and static loads. Therefore, in order to give full play to the anchorage effect of bolts (cables), it is necessary to ensure that the rock mass in zone I has a certain bearing capacity while destressing by excavating the large destressing hole. The buffer zone II provides buffer compensation space for that coal mass in the deep domain of the roadway side continuously migrates to the anchorage surrounding rock. Excavating the large destressing hole within a certain range of the roadway sides will shift stress peak domain of the roadway side to zone III (solid coal side of destressing hole). The stress peak and stress peak places in Zone III play an important role in the maintenance of the roadway.
② When *L*_h_ is small (4–7 m), the rock mass in zone I bears weak stress, the stress value is lower than the in-situ stress value (17.2 MPa), and the bearing capacity is poor. Moreover, owing to the small *L*_h_ in this range, the shift effect of the stress peak place is poor. With the increase in *L*_h_ (from 8 to 10 m), zone I is divided into zones I_a_ and I_b_, according to the different stress states in zone I. There is a high stress domain in zone I_b_. However, the stress in zone I_a_ remains low. Most of the rock mass stress in zone I is less than the in-situ stress and is in a low-stress state. When *L*_h_ is 10 m, the range of the high stress domain in zone I_b_ expands to the shallow surrounding rock. The stress peak in the high stress domain is 19.9 MPa, which is approximately 1.16 times of the in-situ stress. This value is almost equal to the in-situ stress. It can be seen that when *L*_h_ is 10 m, the stress of most of the rock mass in zone I is equivalent to the in-situ stress value.
③ With a further increase in *L*_h_ (11 m → 13 m), the range of zone I_b_ continues to expand to the shallow surrounding rock, and the stress value continues to increase. When the *L*_h_ is 11 m, the stress peak in zone I_b_ is 22.87 MPa, which is about 0.85 times of the previous stress peak. This value is almost equivalent to the previous stress peak, causing a destressing effect that is not obvious. When *L*_h_ is 12 m, the stress peak in zone I_b_ is 25.46 MPa, which is about 0.95 times of the previous stress peak, almost resulting in a loss of destressing capacity. When *L*_h_ is 13 m, the stress peak in zone I_b_ is 27.92 MPa, which is approximately 1.04 times the previous stress peak, which causes ineffective destressing.

(2) Distribution of stress influence curve of large destressing hole place on destressing effect

**Figure 10:**
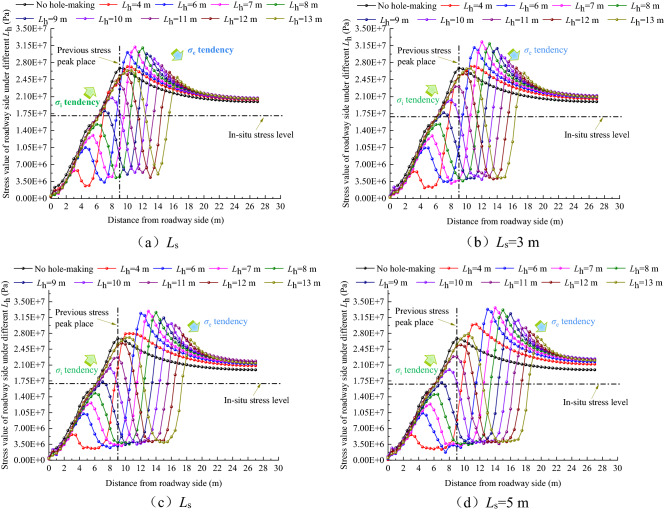
Curve of stress distribution of roadway side at different *L*_h._

The stress distribution curve of the roadway side under different *L*_h_ values is shown in Fig. 10 when *L*_s_ is 2, 3, 4, and 5 m.

As shown in Fig. 10, after the large destressing hole is completed, the stress on the roadway sides is arranged in an asymmetric bimodal shape. With an increase in *L*_h_, the first stress peak (*σ*_i_) gradually increases and continues to approach the previous stress peak (*σ*_o_), and its place (*L*(*σ*_i_)) also approaches the previous stress peak place (*L*(*σ*_o_)). The second stress peak (*σ*_e_) gradually decreases, and its peak place (*L*(*σ*_e_)) continues to be far away from the previous stress peak place (*L*(*σ*_o_)). In addition, the distribution curve of the roadway side stress, with changing *L*_h_, is almost the same under different *L*_s_. Considering the limited length of the paper, this study only discusses in detail the distribution curve of roadway side stress with *L*_h_ changing under 5 m *L*_s_.

① When the *L*_h_ range is between 4 and 7 m, *σ*_i_ is much lower than *σ*_r_. When *L*_h_ is 4, 6, and 7 m, *σ*_i_/*σ*_r_ is 0.33, 0.6, and 0.72, respectively. *σ*_e_ is slightly higher than *σ*_o_. When *L*_h_ is 4, 6, and 7 m, the *σ*_e_ place (*L*(*σ*_e_)) only shifts to the depth of the surrounding rock by 2.5, 4, and 5 m, respectively. It can be seen that excavating the large destressing hole within the *L*_h_ range of 4 to 7 m will not only cause a poor effect of the shift of the high stress peak domain to the deep surrounding rock, but also destroy the integrity of the shallow surrounding rock, resulting in the poor bearing capacity of the shallow surrounding rock, which greatly affects the anchorage effect of the bolts (cables).
② When the *L*_h_ range is between 8 and 10 m,* σ*_i_ increases continuously. When the *L*_h_ is 8, 9 and 10 m, *σ*_i_/*σ*_r_ is 0.85, 0.99, 1.16, and *σ*_i_/*σ*_o_ is 0.54, 0.64, 0.74, respectively. Compared with *σ*_o_, when *L*_h_ is 8, 9, and 10 m, *σ*_i_ is reduced by 46%, 36%, and 26%, respectively. *σ*_e_ decreases with an increase in *L*_h_, and when *L*_h_ is 8, 9, and 10 m, the *σ*_e_ place (*L*(*σ*_e_)) is shifted to the deep surrounding rock by 6, 7, and 8 m, respectively. Combined with the stress nephogram analysis in Fig. 9, it can be seen that excavating the large destressing hole within the *L*_h_ range of 8 to 10 m, especially when the large destressing hole place is 1 m outside the previous stress peak place, can not only realize the effective shift of the high stress peak domain to the deep surrounding rock, but it also does not affect the stress level of the surrounding rock in the anchorage domain. The stress of most rock masses in zone I is equivalent to the in-situ stress value, providing favorable conditions for bolts (cables) anchorage.
③ When *L*_h_ is 11 and 12 m, *σ*_i_/*σ*_o_ is 0.85 and 0.95, respectively. When *L*_h_ is 11 and 12 m, *σ*_i_ is only reduced by 15% and 5%, respectively, when compared with *σ*_o_. When *L*_h_ is 13 m, *σ*_i_ is increased by 1.06 MPa, when compared with *σ*_o_. When *L*_h_ is 11, 12, and 13 m, the distance between the *σ*_i_ place and the *σ*_o_ place (*L*(*σ*_i_) − *L*(*σ*_o_)) is 0, 0.5 and 1.5 m respectively. Excavation of the large destressing hole within the *L*_h_ range of 11 to 13 m will cause *σ*_i_ to be in a high state, and the *σ*_i_ place (*L*(*σ*_i_)) is almost the same as the *σ*_o_ place (*L*(*σ*_o_)), resulting in ineffective destressing.

Through the statistics and analysis of the dynamic change indexes in Fig. 10, Table 5 is obtained.

**Table 5:** Summary of dynamic change indexes of chamber surrounding rock under different *L*_h._

*L*_h_/m	*L*_h_ − *L*(*σ*_o_)/m	*σ*_i_/MPa	*σ*_i_/*σ*_r_	*σ*_i_/*σ*_o_	*L*(*σ*_i_)/m	*L*(*σ*_i_) − *L*(*σ*_o_)/m	*σ*_e_/MPa	*σ*_e_/*σ*_o_	*L*(*σ*_e_) − *L*(*σ*_o_)/m
4	− 5	5.63	0.33	0.21	3	− 6	30.04	1.12	2.5
6	− 3	10.4	0.6	0.39	4.5	− 4.5	33.3	1.24	4
7	− 2	12.4	0.72	0.46	5.5	− 3.5	33.64	1.25	5
8	− 1	14.54	0.85	0.54	6.5	− 2.5	33.18	1.23	6
9	0	17.16	0.99	0.64	7	− 2	32.33	1.20	7
10	1	19.9	1.16	0.74	8	− 1	30.92	1.15	8
11	2	22.87	1.33	0.85	9	0	29.22	1.09	9
12	3	25.46	1.48	0.95	9.5	0.5	28.35	1.06	9.5
13	4	27.92	1.62	1.04	10.5	1.5	27.18	1.01	10.5

**Figure 11:**
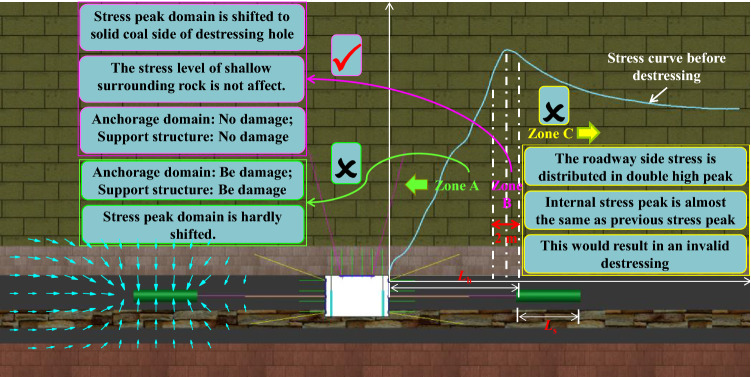
Schematic of *L*_h_ zoning.

When the large destressing hole place is located in the stress peak domain with a 2 m length range, especially when the large destressing hole place is 1 m outside the previous stress peak place, the previous high stress peak domain of the roadway sides can be shifted to the deep surrounding rock without affecting the stress level of the shallow surrounding rock. At the same time, it provides favorable conditions for bolt (cable) anchorage, resulting in good destressing effect, as shown in zone B in Fig. 11 (the range where the previous stress peak place extends 1 m to the inside and outside).

When the large destressing hole place is located inner side this stress peak domain with a length of 2 m, it is easy to destroy the integrity of the shallow surrounding rock and make the shallow surrounding rock mass lose its bearing capacity. Also, the stress shift effect is not obvious, resulting in poor destressing effect, as shown in zone A in Fig. 11.

When the large destressing hole place is located outside the stress peak domain whose length is 2 m, the first stress peak is almost the same as the previous stress peak and the first stress peak place is almost the same as the previous stress peak place, resulting in invalid destressing, as shown in zone C in Fig. 11.

#### Determination of large destressing hole length

1. Comparative analysis of the influence of large destressing hole length on the destressing effect using the stress nephogram

**Figure 12:**
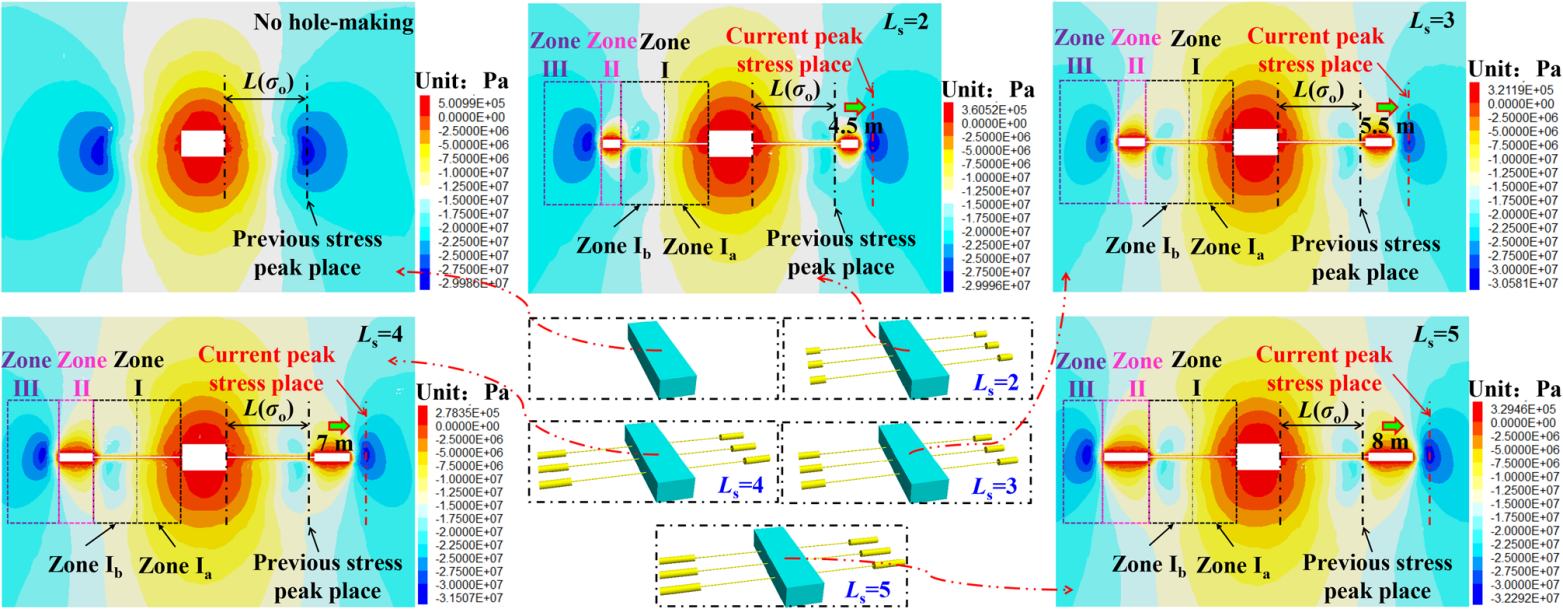
Nephogram of stress distribution of surrounding rock at different *L*_s._

Taking *L*_h_ of 10 m as an example, the nephogram of the stress distribution of the surrounding rock under different *L*_s_ is shown in Fig. 12.

As can be seen from Fig. 12, with an increase in *L*_s_, the volume of the buffer zone (zone II) increases, and the ability to absorb deformation and dynamic load stress wave is improved. The stress peak place in the high stress migration zone (zone III) continues to shift to the deep surrounding rock, and the increase rate of the stress peak is small. The range of the zones I_a_ and I_b_ in the anchorage bearing zone (zone I) almost does not change as *L*_s_ changes.

(2) Distribution of stress influence curve of large destressing hole length on the destressing effect

**Figure 13:**
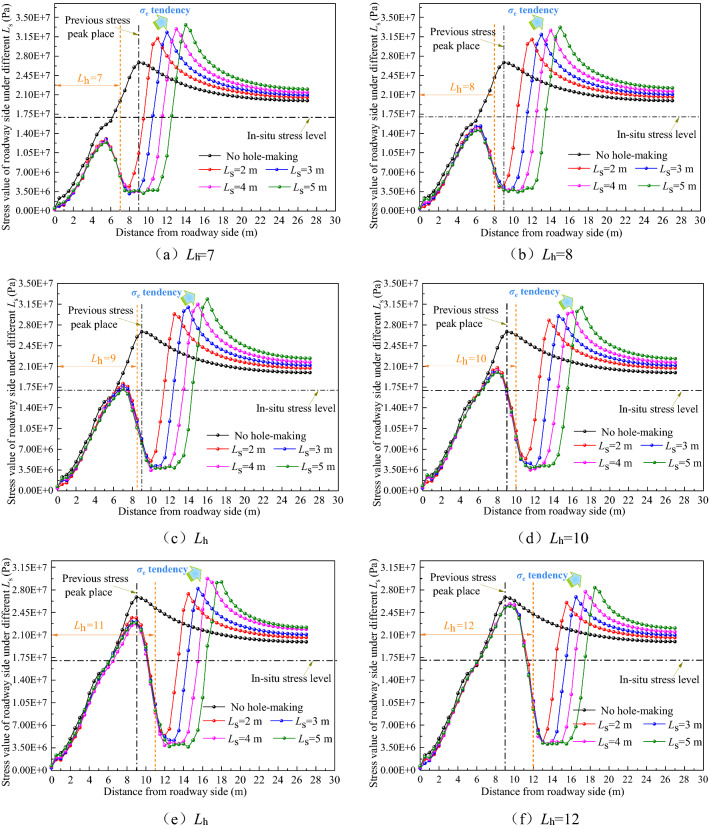
Curve of stress distribution in roadway side rock at different *L*_s._

The stress distribution curve of the roadway side under different *L*_s_ is shown in Fig. 13 for *L*_h_ values of 7, 8, 9, 10, 11, and 12 m, respectively.

① The change in *L*_s_ will not lead to a change in stress inside the large destressing hole place. In other words, the change in *L*_s_ does not change the *σ*_i_ and *σ*_i_ place (*L*(*σ*_i_)), but only change the *σ*_e_ and *σ*_e_ place (*L*(*σ*_e_)), and the volume of the buffer zone (zone II).
② Considering the limited length of the paper, the paper only discusses in detail the distribution curve of roadway side stress under 10 m* L*_h_. When the *L*_s_ is 2, 3, 4 and 5 m, *σ*_e_ is 28.8, 29.47, 30.14, and 30.92 MPa, respectively. The increase gradient of *σ*_e_ is as follows: 2 m → 3 m (2.33%), 3 m → 4 m (2.27%), and 4 m → 5 m (2.59%). When *L*_s_ = 2, 3, 4, and 5 m, the *σ*_e_ place (*L*(*σ*_e_)) is shifted to the deep surrounding rock by 4.5, 5.5, 7, and 8 m, respectively. The increase gradient of the *σ*_e_ place (*L*(*σ*_e_)) shift effect is as follows: 2 m → 3 m (22.22%), 3 m → 4 m (22.27%), and 4 m → 5 m (14.29%). It can be seen that when *L*_s_ increases, the increase gradient of *σ*_e_ is small, but the *σ*_e_ place (*L*(*σ*_e_)) shift effect increases significantly.

Through the statistics and analysis of the dynamic change indexes in Fig. 13, Table 6 is obtained.

**Table 6:** Summary of dynamic change indexes of chamber surrounding rock under different *L*_s._

*L*_s_/m	*σ*_i_/MPa	*L*(*σ*_i_)/m	*L*(*σ*_i_) − *L*(*σ*_o_)/m	*σ*_e_/MPa	*σ*_e_/*σ*_o_	∇(*σ*_e_)/%	*L*(*σ*_e_) − *L*(*σ*_o_)/m	∇[*L*(*σ*_e_) − *L*(*σ*_o_)]/%
2	20.75	8	− 1	28.8	1.07	–	4.5	–
3	20.32	8	− 1	29.47	1.1	2.33	5.5	22.22
4	20.32	8	− 1	30.14	1.12	2.27	7	27.27
5	19.9	8	− 1	30.92	1.15	2.59	8	14.29

It can be seen from Table 6 that the *σ*_i_ value and its places (*L*(*σ*_i_)) are determined by *L*_h_, and the change in *L*_s_ does not lead to a change in stress inside the large destressing hole place. When *L*_s_ increases, the increasing gradient of *σ*_e_ is small, but the shift effect of the *σ*_e_ place (*L*(*σ*_e_)) increases significantly. Therefore, a reasonable increase in *L*_s_ can expand the volume of the buffer zone (zone II) and improve its ability to migrate the high stress peak domain to a deeper surrounding rock.

From the above discussions, it can be seen that on the premise of ensuring the correct large destressing hole place and ensuring a good mechanical environment of shallow surrounding rock, *L*_s_ should be reasonably increased according to the conditions of the on-site construction to improve ability of migrating the high stress peak domain to a deeper surrounding rock. This provides a buffer space for that coal mass in the deep domain of the roadway side continuously migrates to the anchorage surrounding rock.

#### Determination of the large destressing hole spacing

**Figure 14:**
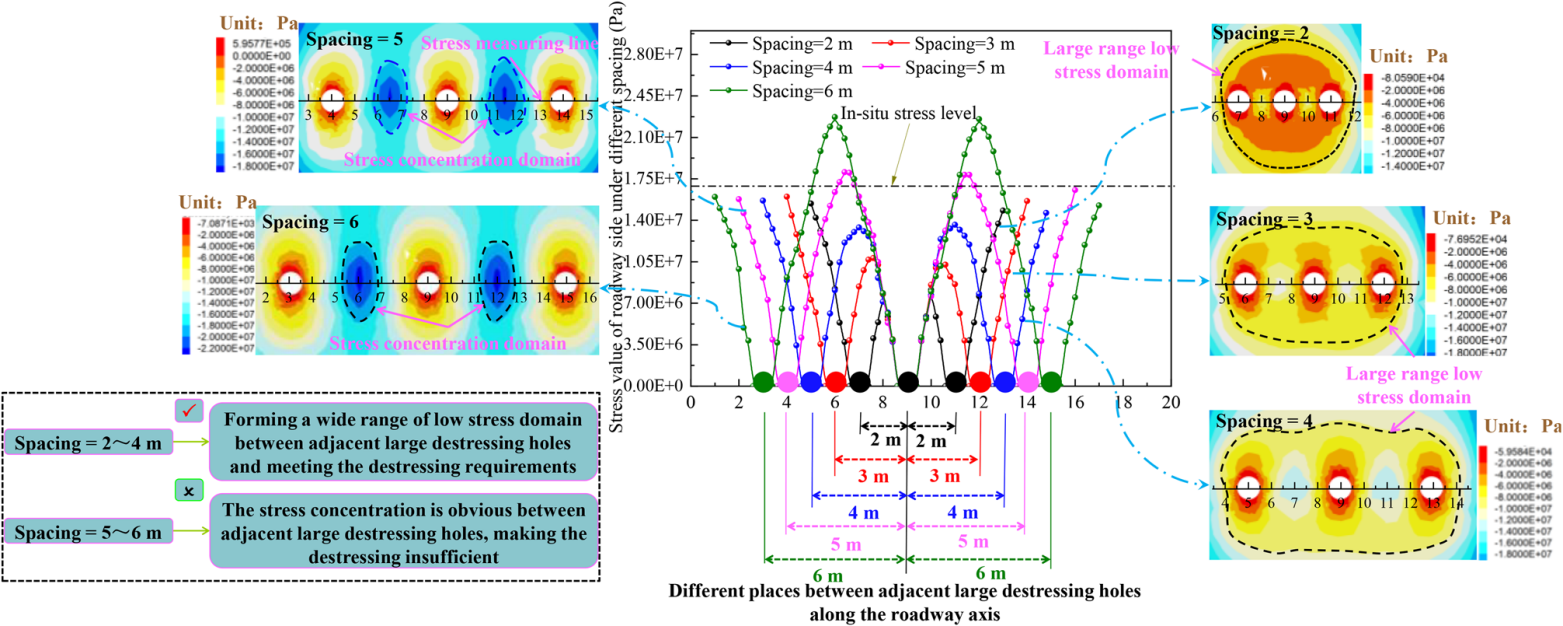
Curve and nephogram of stress distribution of roadway surrounding rock at different spacing.

The stress distribution nephogram and curve of the surrounding rock under different large destressing hole spacings are shown in Fig. 14.

The proper large destressing hole place (*L*_h_) can shift the previous high stress peak domain of the roadway sides to the deep surrounding rock without affecting the stress level of the shallow surrounding rock. Increasing the large destressing hole length (*L*_s_) can expand the volume of the buffer zone (zone II) and improve its ability to migrate the high stress peak domain to a deeper surrounding rock. Therefore, on the basis of reasonably accurate determination of *L*_h_ and *L*_s_, shortening the large destressing hole spacing can release the high abutment pressure sufficiently.

When the spacing between the large destressing holes is between 5 and 6 m, there is an obvious high stress domain between the adjacent large destressing holes, and the stress value in this domain exceeds in-situ stress value. In particular, when the spacing is 6 m, the stress peak reached 22.8 MPa. The large concentrated stress makes the destressing between the adjacent large destressing holes invalid. When the spacing is between 2 and 4 m, owing to the small spacing, the low stress domains generated by single large destressing hole are connected to each other to form successive stress low-value domains in deep surrounding rock, which can ensure that the stress level of the shallow surrounding rock is not impacted and releases the high abutment pressure sufficiently between the deep adjacent large destressing holes. However, considering the progress of the construction and economic benefits, the spacing should not be too small.

Based on the above study, the influence degree of the different large destressing hole parameters on the destressing effect of the roadway surrounding rock is summarized as follows: influence degree of large destressing hole place (*L*_h_) > influence degree of large destressing hole length (*L*_s_) > influence degree of large destressing hole spacing. Based on the geological conditions of the test roadway surrounding rock, it is proposed that the final formation *L*_h_ of large destressing hole on the both sides of the test roadway is 10 m, the large destressing hole length (*L*_s_) is 5 m, and the large destressing hole spacing is 4 m.

## Engineering practice

1. Shallow supporting parameters

The large destressing hole is implemented on the basis of the previous supporting mode. Therefore, the shallow supporting parameters are consistent with "Previous supporting mode and roadway failure".

(2) Deep destressing parameters

The destressing by hole-making technology inside the roadway two sides uses hydraulic hole-making equipment to relieve the pressure. Hydraulic hole-making equipment is mainly composed of crawler hydraulic drill, clean water pump station, vibrating screen solid–liquid separator, high-pressure sealing triangular drill pipe, high and low pressure converter, etc. The above equipments can adapt to a variety of complex geological conditions after long-term development.

Construction sequence: drill a small-diameter hole in the shallow surrounding rock → placing steel pipes into small holes → grouting outside the pipe wall → excavating deep destressing holes → discharging cinders → hole sealing.

**Figure 15:**
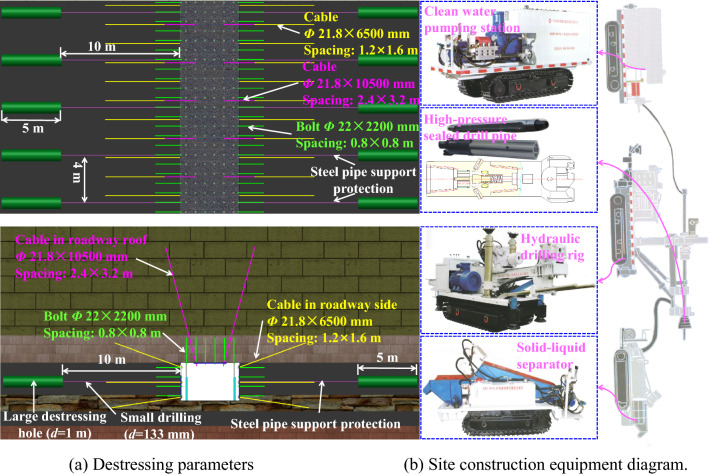
Destressing parameters and site construction equipment diagram.

The final formation *L*_h_ of the large destressing hole on the both sides of the test roadway is 10 m, the large destressing hole length (*L*_s_) is 5 m, and the large destressing hole spacing is 4 m. The shallow borehole diameter was approximately 133 mm. A steel pipe with a diameter of 127 mm is inserted into the shallow small diameter borehole with a diameter of 133 mm, and grouting is carried out outside the pipe wall. The cinder discharge volume of the single deep destressing hole is about 4 m^3^. After the construction of a destressing hole, the shallow small hole shall be sealed with cement. The destressing method and the process parameters are shown in Fig. 15.

**Figure 16:**
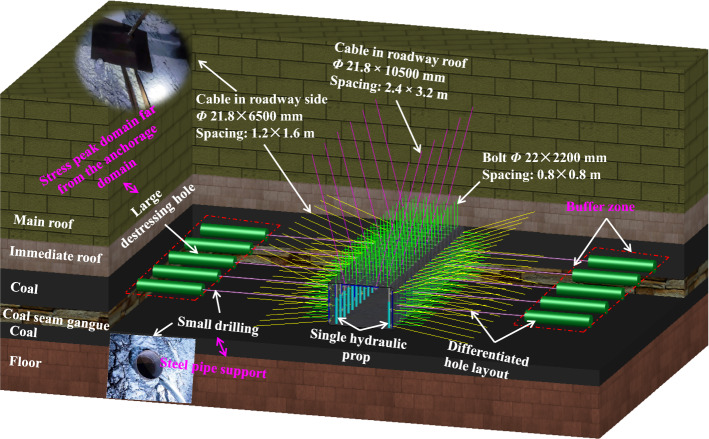
Three-dimensional drawing of comprehensive roadway protection technical scheme.

A stereoscopic view of the technical scheme for comprehensive roadway protection is shown in Fig. 16.

(3) Observation of surrounding rock deformation and anchor cable stress

**Figure 17:**
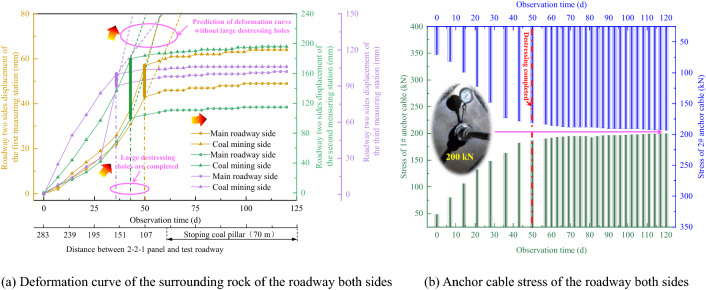
Deformation curve of surrounding rock of both sides and anchor cable stress.

Deformation curve of the surrounding rock of the roadway both sides before and after destressing for the test roadway is shown in Fig. 17a.

The three vertical lines in Fig. 17a represent the actual completion time of the large destressing hole at each measuring station. The large destressing hole construction of the first, second and third measuring stations was completed on the 36th, 43rd and 50 day of field observation, respectively. It can be seen from Fig. 17a that under the influence of the mining turbulence of the 2-2-1 panel, the displacement rate of the surrounding rock on the both sides of the roadway increases significantly before destressing. According to the predicted fitting curve of the change in displacement of the both sides of the roadway without destressing, the roadway width is significantly reduced. When the destressing for excavating a large destressing hole is completed, the displacement rate of the surrounding rock on the both sides of the roadway decreases significantly. When the 2-2-1 panel is advanced near the stopping line (70 m from the roadway), the displacement rate of the both sides of the roadway tends to be stable, and the displacement no longer increases. After the destressing technology is adopted, the stress of the anchor cable tends to flatten obviously, and the stress of the anchor cable does not increase approximately. The stabilized stress of the anchor cable is about 200 kN (see Fig. 17b). Therefore, it is concluded that destressing technology of excavating large destressing hole has a significant effect on controlling the continuous large deformation of the roadway both sides.

## Conclusion

This study proposes an innovative “shallow supporting and deep destressing” synergism technology, obtaining follow innovative conclusions different from the general destressing technology:

1. When the large destressing hole is located in the stress peak domain with a 2 m length range (the range where the previous stress peak place extends 1 m to the inside and outside), and especially when the large destressing hole place is 1 m outside the previous stress peak place, the high stress peak domain of the roadway sides can be shifted to the deep surrounding rock without affecting the stress level of the shallow surrounding rock.
2. The *σ*_i_ and its place (*L*(*σ*_i_)) are determined by *L*_h_, and the change in *L*_s_ does not lead to a change in stress inside the large destressing hole place. When *L*_s_ increases, the gradient of the increase in *σ*_e_ is small, but the shift effect of the *σ*_e_ place (*L*(*σ*_e_)) increases significantly. Therefore, a reasonable increase in *L*_s_ can expand the volume of the buffer zone (zone II) and improve its ability to migrate the high stress peak domain to a deeper surrounding rock.
3. Based on reasonably accurate determination of *L*_h_ and *L*_s_, shortening the large destressing hole spacing can release the high abutment pressure sufficiently. When the large destressing hole spacing is more than 5 m, high stress domain appeared between the adjacent large destressing hole. When the large destressing hole spacing is between 2 and 4 m, the low-stress zones generated by single large destressing hole are connected with each other to form successive stress low-value domains in deep surrounding rock, which can ensure that the stress level of the shallow surrounding rock is not impacted and releases the high abutment pressure sufficiently between the deep adjacent large destressing holes.
4. The influence degree of different large destressing hole parameters on the destressing effect of roadway surrounding rock is obtained as follows: influence degree of large destressing hole place > influence degree of large destressing hole length > influence degree of large destressing hole spacing. The engineering practice shows that the “shallow supporting and deep destressing” technology has a significant effect on controlling the continuous large deformation of the both sides of the roadway.

## Data Availability

The datasets used and/or analysed during the current study available from the corresponding author on reasonable request.
